# American Medical Association—Section on Dental and Oral Surgery

**Published:** 1896-05

**Authors:** 


### NOTICES.

### American Medical Association—Section on Dental and Oral Surgery.

1. Chairman's Address, R. R. Andrews, Cambridge, Mass.

2. "A Few of the Causes of Failures in the Dental and Medical Professions," B. B. Smith, Pensacola, Fla.

3. "Modern Methods of Treating the Antrum of Highmore," W. X. Sudduth, Chicago, Ill.

4. "Further Investigations Upon the Antrum," M. H. Fletcher, Cincinnati, Ohio.

5. "Cataphoresis," H. W. Gillett, Newport, R. I.

6. "The Technique and Pathology of the Peridental Membrane," Vida A. Latham, Chicago, Ill.

7. "Movements of the Mandibular Condyles and Dental Articulation," W. E. Walker, Pass Christian, Miss.

8. "Disease of the Oral Cavity, a Potent Factor of General Disease," S. W. Foster, Atlanta, Ga.

9. "Treatment of Children During the Period of Dentition," H. H. Johnson, Macon, Ga.

10. "Professional Congeniality," H. D. Wilson, Bainbridge, Georgia.

11. "The Replacement of the Superior Maxilla by a Mechanical Appliance," Thos. P. Himman, Atlanta, Ga.

12. "Practical Illustrations in Conservative Surgery," G. Lenox Curtis, New York City.

13. "——————," G. V. I. Brown, Duluth, Minn.

14. "Pyorrhœa Alveolaris," Eugene S. Talbot, Chicago.

